# Evaluation of riverine macro- and mesoplastic monitoring approaches

**DOI:** 10.1007/s10661-025-14889-4

**Published:** 2026-01-16

**Authors:** Stephanie B. Oswald, Paul Vriend, Ad M. J. Ragas, Margriet M. Schoor, Frank P. L. Collas

**Affiliations:** 1 Department of Environmental Science, Radboud Institute for Biological and Environmental Science (RIBES), Radboud University, P.O. Box 9100, 6500 GL Nijmegen, The Netherlands; 2 Rijkswaterstaat, Ministry of Infrastructure and Water Management, The Hague, The Netherlands; 3 Institute of Environmental Sciences, Leiden University, Leiden, The Netherlands

**Keywords:** SWOT analysis, Freshwater systems, Monitoring techniques, Plastic pollution, Net sampling

## Abstract

Globally, plastic pollution in aquatic environments has become an emerging concern. A multitude of monitoring techniques have been used to collect information on the presence of macroplastics (> 25 mm) and mesoplastics (> 5 mm ≤ 25 mm) in river systems. The differences between these methodological approaches, combined with limited knowledge of the fundamental processes that affect plastic presence and detection ability, lead to a lack of standardization between methods and limit comparisons of quantitative data on plastic pollution among studies. This study investigates how different monitoring approaches, e.g., larvae net, trawl net, and stow net, affect the observed abundance and composition of macro- and mesoplastics in the Rhine-Waal Rivers. Additionally, we performed a SWOT analysis highlighting the strengths, weaknesses, opportunities, and threats of the three methods. During trawl net and stow net monitoring, more unique macro- and mesoplastics categories were found in comparison with simultaneous larvae net monitoring. However, while mean macroplastic concentrations were higher in the stow net samples (1.22 × 10^–3^ items/m^3^) than in the larvae net (0.91 × 10^–3^ items/m^3^), mesoplastics concentrations were significantly higher in the larvae net samples (6.91 × 10^–3^ items/m^3^) compared to those recorded with the stow net (1.15 × 10^–3^ items/m^3^), indicating that the size plastic recovery rates depend on the sampling technique. The SWOT analysis pointed towards a better overall performance of the trawl net. The outcome of the current study can be used to support policymakers, industry, and the scientific community in developing successful monitoring strategies for macro- and mesoplastics pollution in rivers. The SWOT analysis may facilitate the choice of the approach that best aligns with the specific monitoring goals and the environmental conditions of the target area.

**Supplementary Information:**

The online version contains supplementary material available at 10.1007/s10661-025-14889-4.

## Introduction

Plastic pollution is a significant environmental challenge facing the world today (Roebroek et al., 2021; Winton et al., 2020). The negative impacts of plastic pollution on the environment have been widely discussed (Fossi et al., 2019; Proshad et al., 2018; Welden, 2020). While adverse effects have been well-documented in marine systems (e.g., Thushari & Senevirathna, 2020), an increasing number of studies report environmental impacts of similar magnitude in freshwater systems (Roebroek et al., 2021; van Emmerik et al., 2022a). Despite the growing understanding of plastic sources (Li et al., 2023; Oswald et al., 2023; Strokal et al., 2021), fate and behavior (Nakayama & Osako, 2023; Oswald et al., 2025; Vriend et al., 2023), and ecological consequences of plastics in riverine systems (Gall & Thompson, 2015; Macali et al., 2018; Al-Zawaidah et al., 2021), the potential impacts and implications of plastics on the riverine environment deserve more scientific attention, as rivers provide important societal functions (Postel, 2000). These include, among others, habitat for aquatic life, irrigation for agriculture, source of water for human consumption, inland navigation, and recreation (Jackson et al., 2001; Lebreton et al., 2017; Richter et al., 2003). Moreover, scientific research indicates that a substantial proportion of plastics do not undergo transport to marine ecosystems but are retained in the river system, underscoring the significance of studying plastic pollution in rivers (van Emmerik et al., 2022a). Therefore, understanding the magnitude and dynamics of plastic pollution in rivers requires consistent and well-designed monitoring efforts.

Monitoring can be defined as continuous and regular observations of environmental characteristics or processes to collect data and identify spatial or temporal trends (Artiola et al., 2004; GESAMP, 2019), thereby providing the basis for assessing the nature and extent of plastic pollution in rivers, and for the design of appropriate mitigation measures, as well as evaluating their effectiveness (GESAMP, 2019; van Emmerik et al., 2022b). Field studies conducted worldwide have developed and used a range of methods to quantify and describe plastic pollution in riverine environments, including visual observations (i.e., González-Fernández & Hanke, 2017; Vriend et al., 2020), remote sensing technologies (i.e., Flores et al., 2022; Geraeds et al., 2019; Sakti et al., 2023), and sampling nets (i.e., Morritt et al., 2014; Oswald et al., 2023; Tokai et al., 2021). This plethora of available sampling methods, combined with the variety of specific compartments sampled by each technique, has led to a lack of standardization (Hurley et al., 2023). Additionally, an assessment of the degree to which the quantification of plastic pollution is affected by the used monitoring methodology is lacking. Subsequently, the comparability of plastic pollution levels between studies and regions is inherently flawed (Gallitelli et al., 2020).

The decision on the appropriate method to sample and monitor plastic in the river will depend on several factors (Brander et al., 2020). For instance, the research objectives, which will determine the type and amount of data required; available resources, including time, personnel, equipment, and funding; and the characteristics of the river being studied, such as flow rate, water depth, and width (Ryan et al., 2020; Vrana et al., 2005; Vriend et al., 2020). A SWOT analysis can be a useful tool for assessing the strengths, weaknesses, opportunities, and threats associated with different sampling methods, leading to a more informed decision on the most appropriate plastic monitoring method (Schreyers et al., 2021).

Accordingly, this study discusses and evaluates various techniques for sampling macro- and mesoplastics pollution in the water column of the Rhine River and its major branch, the Waal River. We hypothesize that different sampling techniques vary significantly in their ability to capture macro- and mesoplastics, leading to differences in recovery efficiency and particle characterization. The aim of this study is not to compare absolute levels of plastic pollution detected by different methods but rather to assess how the choice of sampling technique influences the sensitivity and representativeness of plastic recovery. Furthermore, we provide insights into method selection through a SWOT analysis. Overall, the findings of this study provide a scientific basis for selecting appropriate sampling methods that can be used to support policymakers, industry, and the scientific community.

## Methods

### Study area

Sampling campaigns were conducted in the Rhine River and its major branch, the Waal River, in the Netherlands (Appendix A, Fig. A1). The Rhine stretches 1,233 km from its source in Switzerland to the North Sea (ICPR, 2022). Upon entering the Netherlands at Lobith, the Rhine splits into the Waal River and the Pannerdensch Canal. The Waal is the main inland navigation transport route connecting the port of Rotterdam to the German hinterland (Ten Brinke et al., 1999). Two different locations along the Rhine-Waal River were selected for macro- and mesoplastics monitoring (Appendix A, Fig. A1).

**Fig. 1:**
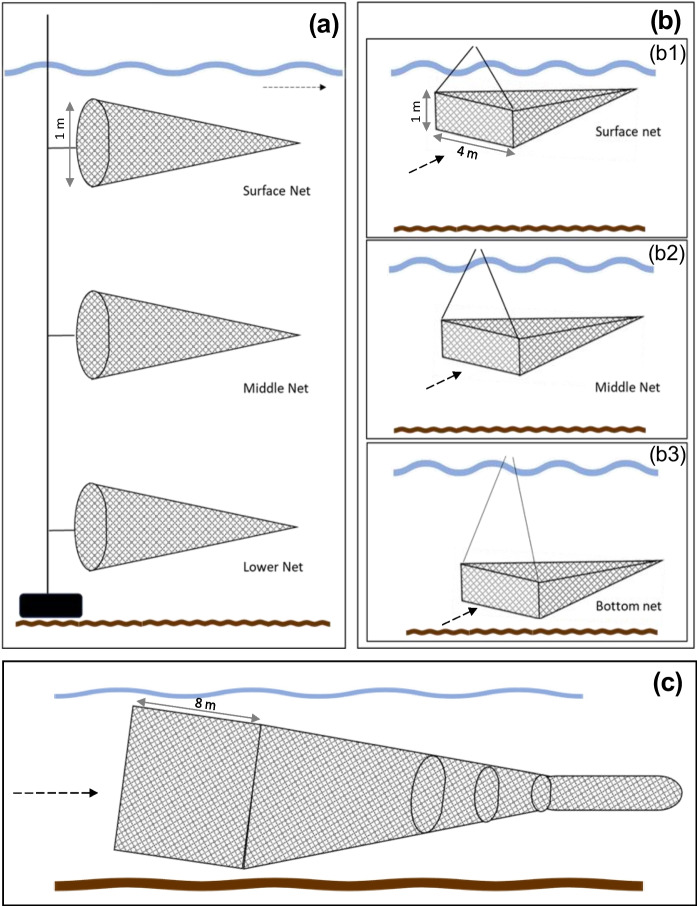
Sketched representation of the plastic monitoring methods: (**a**) larvae net from a side view, (**b**) trawl net, and (**c**) side view of the stow net (Adapted from Oswald et al., 2025)

### Monitoring techniques

The methods used for macro- and mesoplastics sampling in the rivers Rhine and Waal are described below and include three different sampling nets: 1) larvae net, 2) stow net, and 3) trawl net (Table 1; Fig. 1; Collas et al., 2021; Oswald et al., 2023; Vriend et al., 2023). At each location, two monitoring techniques were deployed simultaneously in parallel, under equivalent flowing conditions (i.e., in the Waal River, a stow net and a larvae net; in the Rhine River, a larvae net and a trawl net; Appendix A, Fig. A1). Due to differences in the monitoring techniques configuration, and deployment requirements, the number of samples per method was not directly proportional.

**Table 1:**
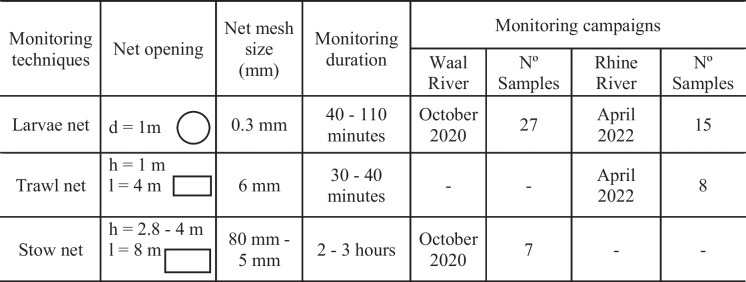
Location and occurrence of parallel monitoring campaigns using different methods: larvae net, trawl net, and stow net, in the rivers Waal and Rhine

#### Larvae net

The larvae net monitoring technique operates with three nets positioned simultaneously in a stacked arrangement at different depths: one positioned just below the water surface, the second placed in the middle of the water column, and the third situated above the riverbed. The three nets were connected to each other and to a weight that ensured their stability by resting on the riverbed (Fig. 1a). Each net had a circular opening with a diameter of 1 m and a mesh size of 0.3 mm. Per sampling day, the net was retrieved 5 times, yielding an average of 15 samples per day. The average monitoring duration was 63 min.

#### Trawl net

The trawl net is deployed at one sampling depth at a time, allowing monitoring at different water depths, sequentially right below the water surface, in the middle of the water column, and right above the riverbed (Fig. 1b). Each net has an opening of 1 × 4 m and a mesh size of 6 mm. The trawl nets were lowered using cranes for 30 to 40 min, resulting in an average of 12 samples per day.

#### Stow net

The stow net is a bag-shaped net that was connected to an anchored fishing boat. The net was held open by two beams, one near the riverbed and a second partially above the surface water, passively sampling plastic pieces throughout the entire water column (Buoyant + Suspended + Bed-Load; Fig. 1c). The stow net had a width of 8 m, while the height ranged from 2.8 m to 4.0 m, varying according to the water level. The mesh size varied from 80 mm, becoming progressively smaller towards the end of the net, with a mesh size of 5 mm. Per sampling day, the net was retrieved between 4 and 5 times. The interval of each sampling session ranged between 2 and 3 h.

### Processing and identifying samples

Across all monitoring campaigns, plastic debris was visually separated from organic matter and fish caught in the onboard net. The collected plastic particles were washed in the same laboratory to remove any remaining organic matter. To prevent loss, the washing process was conducted over a 500-micron sieve. The plastic items were then sorted, counted, and categorized by size: macroplastics (> 25 mm) and mesoplastics (> 5 mm ≤ 25 mm). Finally, all samples were classified according to the River-OSPAR plastic checklist (Schone Rivieren, 2021; Appendix B), allowing for a direct comparison of item categories sampled by each technique. The River-OSPAR protocol was developed by Stichting De Noordzee (SDN) as an adaptation of the OSPAR Guidelines for beach litter monitoring, which was developed by the OSPAR Commission, to make them suitable for riverine environments. The checklist includes a detailed item categorization system, classifying plastics and other litter according to their typical use (van Emmerik et al., 2020).

### Statistical analyses

#### Plastic categories

A 'Rarefaction Curve (RC)' was used to determine the cumulative observed OSPAR categories in relation to the collected number of macro- and mesoplastics items. These curves were used to identify differences in the total number of observed categories using the *“vegan”* package, v. 2.6–4 in R-statistics (R Core Team, 2022; Oksanen et al., 2022). For this purpose, the relative abundance of each collected OSPAR category was determined separately for each monitoring technique (e.g., trawl net, larvae net, and stow net). To determine whether the number of OSPAR categories of macro- and mesoplastics detected differed among sampling methods, a Chi-square test was performed.

Additionally, the diversity of macro- and mesoplastic types (OSPAR categories) collected by different sampling techniques was assessed using the Shannon–Wiener (H′) and Simpson (1–D) diversity indices, number richness, and Pielou's Evenness (“vegan” package in R: v. 2.6–6.1). These indices reflect both diversity, the number of plastic categories (richness), and how evenly they were distributed (evenness) within each sample.

#### Plastic concentration

As there were inherent differences between applied methodologies, the concentrations collected were statistically compared. Prior to the analyses, the distribution of the plastic concentration was determined using the “fitdistrplus” package (Delignette-Muller & Dutang, 2015). Subsequently, due to a gamma-distributed dataset, a generalized linear model (GLM) with a log-link was used to analyze the effect of the “sampling method” (categorical variable) on the concentration of detected macro- and mesoplastics using the larvae net and the trawl net. A linear model (LM) was used for the comparison of found macro- and mesoplastics concentrations between the larvae net and the stow net methods, as these concentrations were found to be normally distributed. Sampling depth was not included in the analyses due to an unbalanced sampling design of the trawl net monitoring and the lack of depth differentiation of the stow net sampling. The analyses were performed using the GLM and LM functions in R statistics (R Core Team, 2019).

#### SWOT analysis

Sixteen factors were chosen to develop a comprehensive multi-criteria decision approach to assist in the creation of the SWOT analysis, including the strengths, weaknesses, opportunities, and threats associated with the distinct methods of plastic monitoring. The key factors were grouped into monetary, adaptability, operational, and limiting factors. Monetary factors encompass daily costs, the minimum number of staff required, and laboratory expenses. Adaptability factors consider whether anchoring is required, the ability to collect data while sailing, the spatial location, and the different depths at which the method can be applied. Operational factors include the width setup of the boat and the net, the number of samples collected per day, and the net mesh size. Lastly, limiting factors include the risk of the net getting clogged, necessary permits, fish and plant bycatch, and selective filtration limitations.

To acquire all data regarding the factors included in the SWOT analysis, government staff, boat employees, and fishermen involved in plastic monitoring campaigns were consulted. The responses obtained are included in Appendix C (Table C1). These results were also used as a basis for constructing a heatmap indicating the applicability of each sampling method.

## Results

### Plastic categories

A total of 2,460 items of plastic were counted, of which 867 were macroplastic items (> 25 mm) and 1,593 mesoplastic items (> 5 mm ≤ 25 mm). During trawl and stow net monitoring, more unique macro- and mesoplastic categories were found than during simultaneous larvae net monitoring (Appendix D). Specifically, 8 unique macroplastic categories were recorded in the trawl net samples, and 10 in the stow net. For mesoplastics, 4 unique categories were recorded in the trawl net samples, and 4 in the larvae net. However, the dominant category types follow the same patterns among methods, where ‘Plastic film 2.5 −50 cm (soft)’ and ‘Plastic film 0—2.5 cm (soft)’ for macro- and mesoplastics, respectively, were the most reported categories collected by all three methods (Appendix D).

**Fig. 2:**
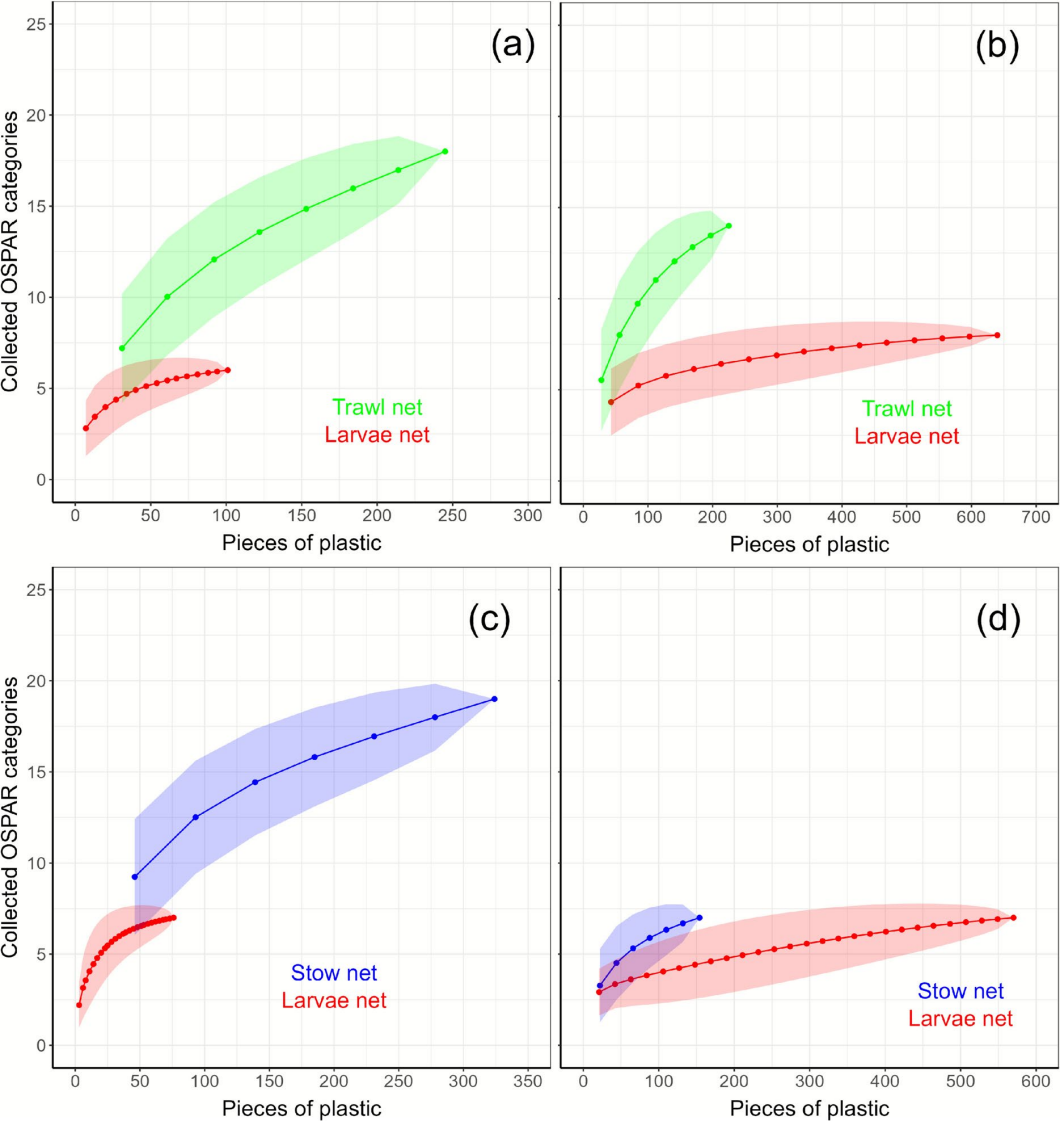
'Rarefaction curve' of the cumulatively observed OSPAR-categories in relation to the collected number of pieces of (**a**, **c**) macroplastics and (**b**, **d**) mesoplastic with the methods: trawl net, larvae net, and stow net

A difference was observed in the number of OSPAR categories in relation to the collected macro- and mesoplastics pieces among the different methods. The trawl net collects more categories than the larvae net, for both macro- and mesoplastics (Fig. 2a, b, respectively; Table 2), indicating that for the same number of collected macroplastic pieces (e.g., 50 pieces), the trawl net collects around nine different categories, whereas the larvae net only collects five. (Fig. 2a). Further differences were also observed when comparing the larvae net and the stow net, as the stow net collects a greater number of categories (Fig. 2c, d, respectively; Table 2). For instance, the 'rarefaction curve’ of the cumulatively observed mesoplastics categories indicates that approximately 550 pieces of mesoplastics need to be collected by the larvae net in order to collect the same number of categories using a stow net, where this only needs 150 pieces to collect 7 different categories (Fig. 2d). In general, the number of categories collected by the larvae net levels off at a lower level compared to the other techniques, implying that the methodology showcases a biased sampling in certain categories.

**Table 2:** Diversity indices, including Shannon–Wiener (H′), Simpson (1–D), richness, and Pielou's Evenness, of (a) macro- and (b) mesoplastics categories collected using trawl and larvae net, and of (c) macro- and (d) mesoplastics categories collected using larvae and stow net

**(a) Diversity indices of macroplastics collected using Trawl and Larvae nets**				
	Shannon–Wiener (H′)	Simpson (1–D)	Richness	Pielou's Evenness
Trawl net	1.512266	0.6170036	19	0.5136008
Larvae net	1.166897	0.6019018	7	0.5996665
**(b) Diversity indices of mesoplastics collected using Trawl and Larvae nets**				
	Shannon–Wiener (H′)	Simpson (1–D)	Richness	Pielou's Evenness
Trawl net	1.0184184	0.4249000	11	0.4247135
Larvae net	0.7676185	0.3521875	8	0.3691465
**(c) Diversity indices of macroplastics collected using Larvae and Stow nets**				
	Shannon–Wiener (H′)	Simpson (1–D)	Richness	Pielou's Evenness
Larvae net	1.41513	0.6890582	7	0.7272329
Stow net	1.559205	0.6177602	19	0.5295422
**(d) Diversity indices of mesoplastics collected using Larvae and Stow nets**				
	Shannon–Wiener (H′)	Simpson (1–D)	Richness	Pielou's Evenness
Larvae net	0.7358019	0.3879224	7	0.3781274
Stow net	0.6471758	0.2688866	7	0.3325826

The difference in the relative abundance of macroplastic categories between the larvae net and trawl net is statistically significant (χ^2^ = 31.996, p < 0.05). Whereas the difference in the relative abundance of mesoplastics categories between methods, despite appearing slightly different, is not statistically significant (χ^2^ = 5.4556, p = 0.941). Comparisons between the larvae net and stow net showed statistically significant differences for both macro- and mesoplastics (χ^2^ = 69.497, p < 0.05; χ^2^ = 17.485, p < 0.05, respectively).

The previously observed differences in macro- and mesoplastics composition among methods were further supported by diversity indices (Table 2). The trawl net exhibited higher overall diversity of macroplastics categories, with a Shannon–Wiener index (H′) of 1.51 and a Simpson's Index (1–D) of 0.62, compared to the larvae net, which showed lower values (H′ = 1.17, 1–D = 0.60). Similarly, category richness was greater in the trawl net (19) than in the larvae net (7). Evenness, however, was slightly higher in the larvae net (0.60) than in the trawl net (0.51), suggesting a more uniform distribution of macroplastic categories in the larvae net samples, despite lower overall diversity and richness. Similarly, for mesoplastics, trawl net samples showed higher diversity and richness (H′ = 1.01, 1–D = 0.42, richness = 11) compared to the larvae net (H′ = 0.77, 1–D = 0.35, richness = 8), although evenness remained comparatively low in both methods.

When comparing the larvae net with the stow net, the stow net captured a greater diversity of macroplastic categories (H′ = 1.56, 1–D = 0.62, and substantially higher richness (19) than the larvae net (H′ = 1.42, 1–D = 0.69, richness = 7). Conversely, evenness was higher in the larvae net (0.73) than in the stow net (0.53). For mesoplastics, both methods recorded equal richness (7), but the larvae net showed slightly higher diversity (H′ = 0.74, 1–D = 0.39) and evenness (0.38) than the stow net (H′ = 0.65, 1–D = 0.27, evenness = 0.33).

### Plastic concentration

**Fig. 3:**
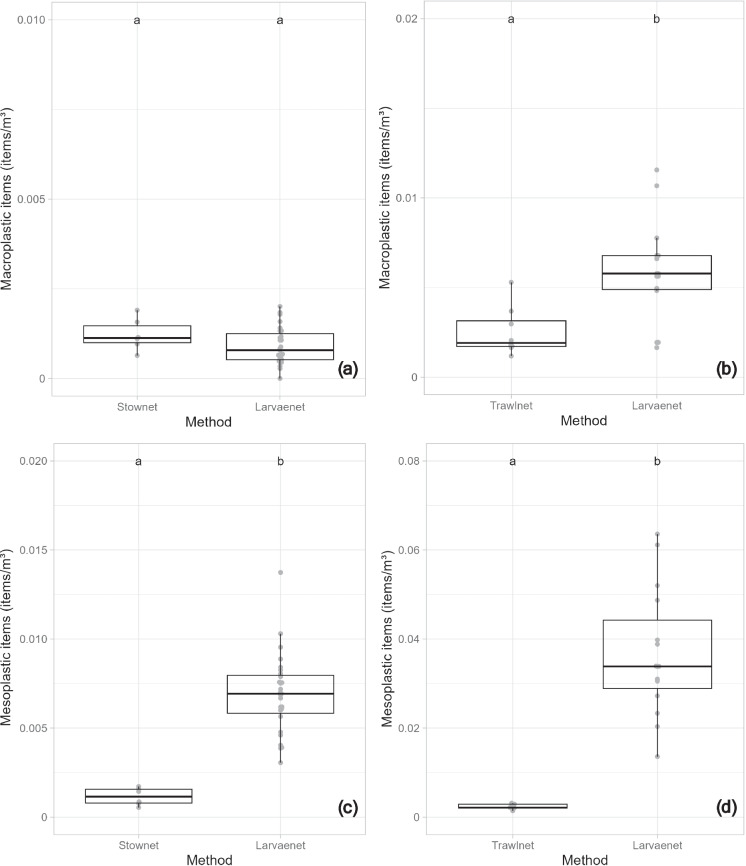
Direct comparison of the effect of each sampling method in the Waal River, e.g., stow net versus larvae net on (**a**) macro- and (**c**) mesoplastics concentrations (items per m^3^), and in the Rhine River, e.g., Trawl net versus larvae net on (**b**) macro- and (**d**) mesoplastics concentrations (items per m^3^). (Different letters depict significant differences). The bands in the middle of the boxes are the median; the lower and upper bands of the boxes are the 25th and 75th percentiles, respectively. The upper and lower whiskers were derived using the standard setting in R statistics, and the dots represent macro- and mesoplastics concentration data

In the Waal River, macroplastic concentrations did not differ significantly between the stow net and larvae net samples (F-value = 1.911, df = 1, p = 0.177). The mean macroplastic concentration was 1.22 × 10^–3^ items/m^3^ for the stow net and 0.91 × 10^–3^ items/m^3^ for the larvae net (Fig. 3a; Appendix E, Table E2). In contrast, mesoplastics concentrations were significantly higher in the larvae net samples compared to the stow net samples (F-value = 37.843, df = 1, p < 0.001), with mean concentrations of 6.91 × 10^–3^ items/m^3^ and 1.15 × 10^–3^ items/m^3^, respectively (Fig. 3c; Appendix E, Table E2).

In the Rhine River, macroplastic concentrations were significantly higher in the larvae net samples compared to the trawl net samples (χ^2^ = 3.311, df = 1, p < 0.001; Nagelkerke R^2^ = 0.41). The mean macroplastic concentration in the Rhine River was 2.54 × 10^–3^ items/m^3^ for the trawl net and 5.88 × 10^–3^ items/m^3^ for the larvae net (Fig. 3b; Appendix E, Table 1). Similarly, mesoplastics concentrations were significantly higher in the larvae net samples than in the trawl net samples (χ^2^ = 25.989, df = 1, p < 0.001; Nagelkerke R^2^ = 0.94), with mean concentration of 2.33 × 10^–3^ items/m^3^ for the trawl net and 36.78 × 10^–3^ items/m^3^ for larvae net (Fig. 3d; Appendix E, Table E1).

### SWOT analysis

**Fig. 4:**
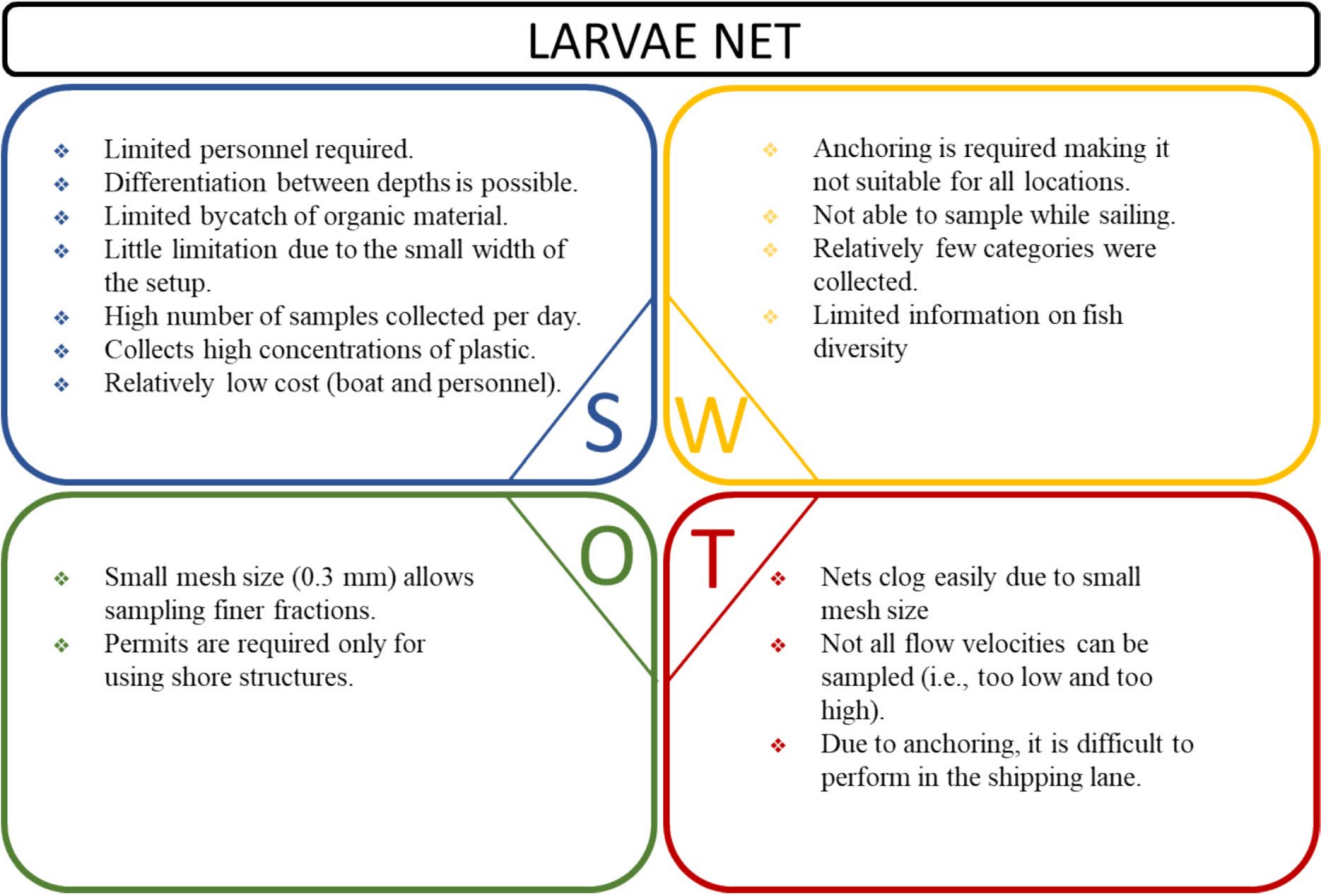
SWOT analysis including the strengths (S), weaknesses (W), opportunities (O), and threats (T) of the larvae net monitoring technique based on application in the rivers Waal and Rhine in October 2020 and April 2021

**Fig. 5:**
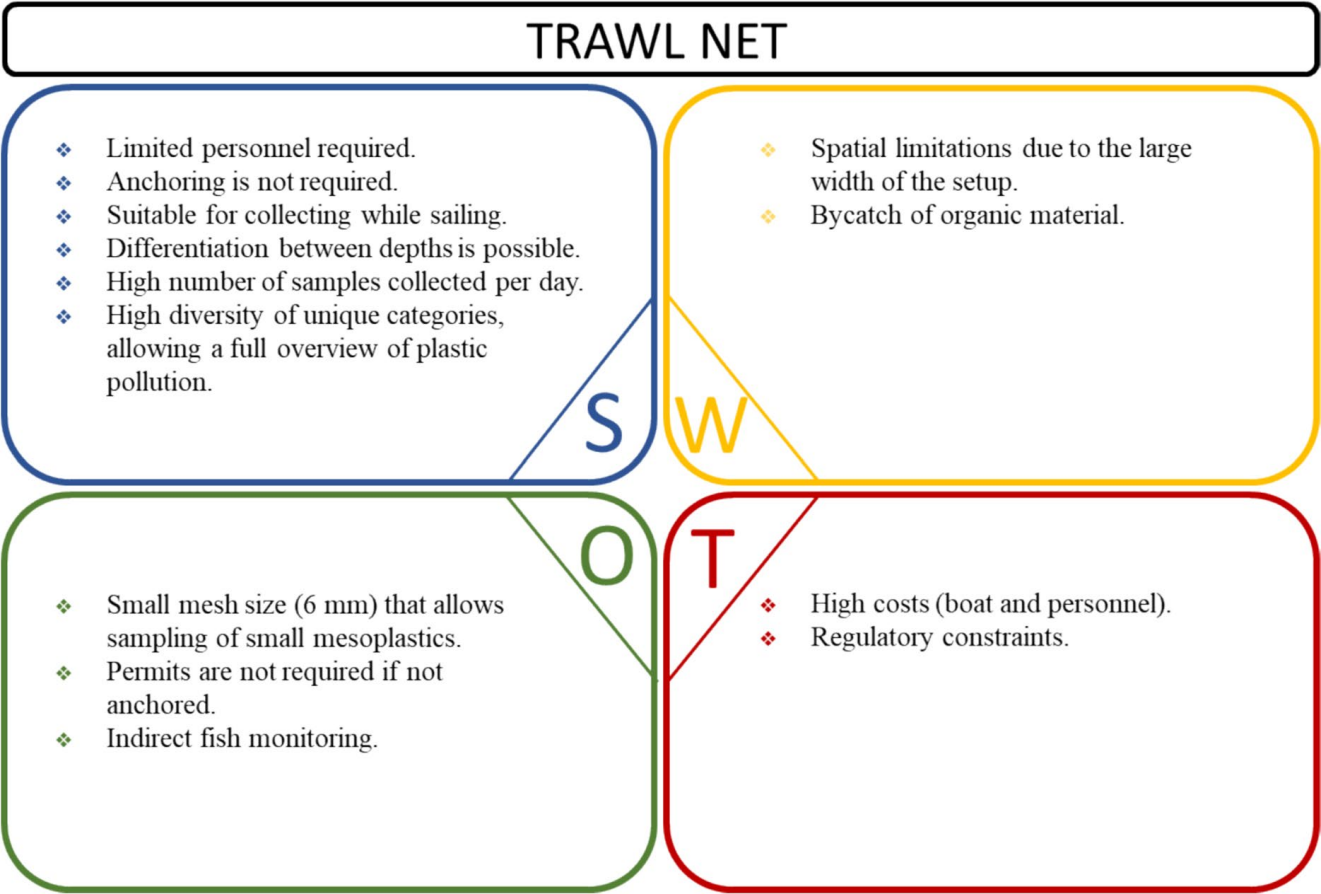
SWOT analysis including the strengths (S), weaknesses (W), opportunities (O), and threats (T) of the trawl net monitoring technique based on application in the Rhine River in April 2021

**Fig. 6:**
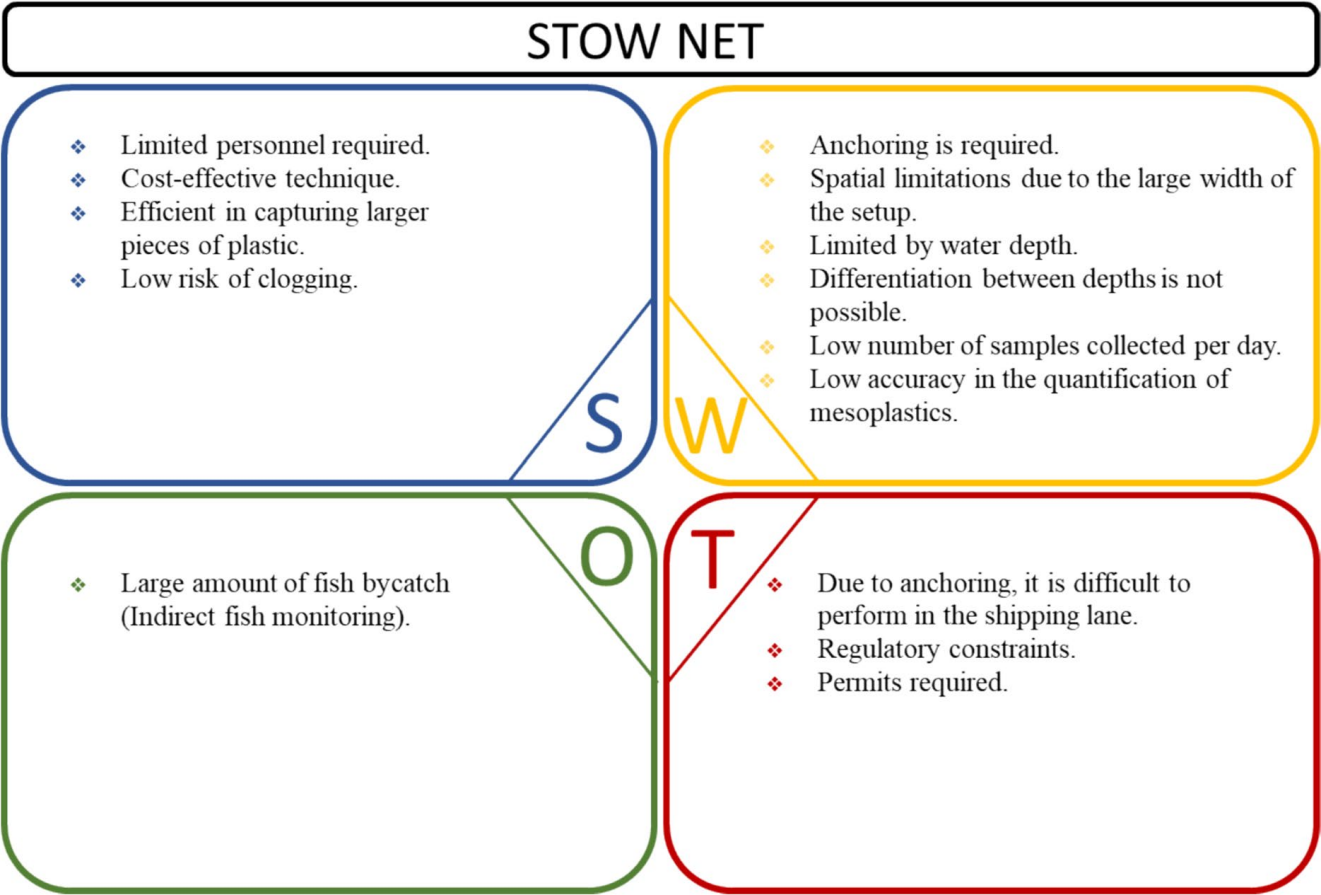
SWOT analysis including the strengths (S), weaknesses (W), opportunities (O), and threats (T) of the stow net monitoring technique based on application in the Waal River in October 2020

**Fig. 7:**
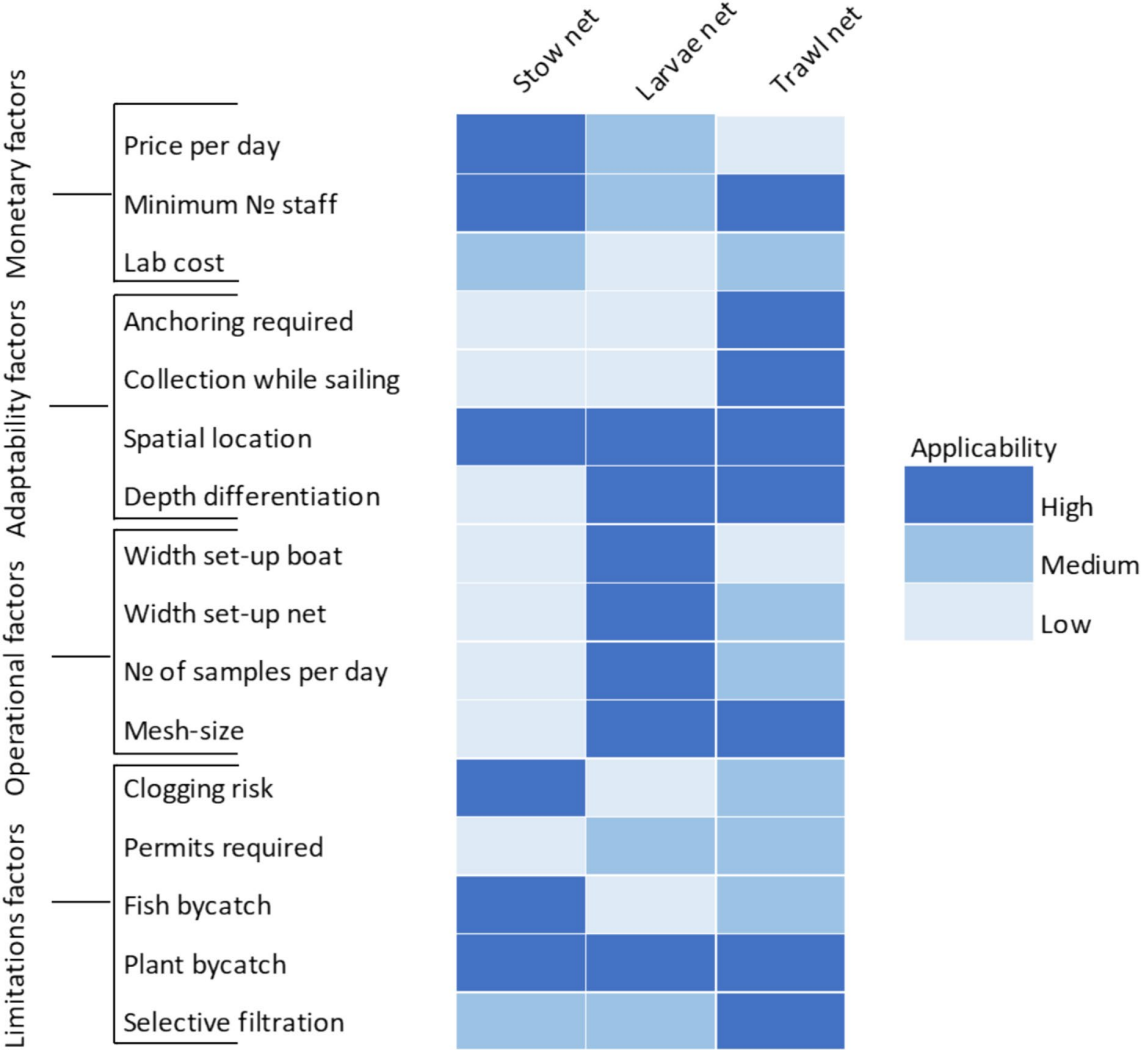
Comparative visualization of the applicability of each plastic monitoring technique across selected factors for developing the SWOT analysis (Appendix C, Table C1)

Figures 4, 5, and 6 represent the SWOT analysis, including the strengths, weaknesses, opportunities, and threats of each distinct method of plastic monitoring (i.e., larvae net, trawl net, and stow net, respectively). A list of 16 factors relevant to each method was used as application indicators for the SWOT analysis (Fig. 7; Appendix C, Table C1).

#### Larvae net

Strengths of the larvae net technique include its operational simplicity and low personnel requirement. In general, the used larvae net was easy to assemble and to place in the water, requiring a minimum of two people. Its design allows for depth-differentiated sampling through simultaneous deployment at multiple depths in a stacked arrangement. This method also results in limited bycatch of organic material, which helps to preserve sample integrity and facilitates the subsequent plastic items extraction. The small-width setup boat facilitates navigation through narrow waterways like rivers, tributaries, and channels, expanding accessibility to sampling locations that may be inaccessible to larger vessels. The high throughput of the method enables a rapid turnaround, allowing for the collection and processing of a significant number of samples per day. The ability to process a large number of samples quickly is often desirable. This method can collect a significantly high concentration of plastic, but the quantification of the sampled volume is potentially flawed.

Despite its strengths, the larvae net technique also has some weaknesses that should be addressed. Anchoring is required for deployment, which restricts its usability in certain locations. The nets are connected to a weight that rests on the bottom to ensure stability, limiting the technique to sampling while sailing. The diversity of plastic categories collected by larvae nets is relatively low. Analysis of the "rarefaction curve" of cumulatively observed categories indicates a smaller number of unique categories sampled by the larvae net in comparison to other sampling methods employed. This suggests that the larvae nets may not be as comprehensive in capturing the full range of plastic pollution in aquatic environments. Furthermore, compared to the other monitoring techniques, the larvae net does not provide any insights into the local fish community, as there is no fish bycatch. The lack of potential applicability to monitor fish reduces the likelihood of co-funding from a fish-monitoring perspective.

The larvae net method also presents valuable opportunities for expanding plastic monitoring efforts. Its fine mesh size (0.3 mm) allows for the selective filtration of smaller particles, including microplastics (< 0.5 mm). Additionally, permits are only required for using shore structures, simplifying regulatory requirements associated with fieldwork.

One significant threat of this technique is the risk of clogging, particularly in areas with high levels of plastic pollution, a large sediment load, or abundant organic material. The larvae net may be prone to obstructions, leading to frequent maintenance to clear and empty the net to ensure continued functionality. Yet, the use of larvae nets can be limited by adverse physical conditions such as flow velocity (too low or too high) and strong currents. These conditions may threaten the ability to constantly collect plastic items. Additionally, the presence of navigation activities in the area may trigger safety concerns and logistical challenges for deploying and retrieving larvae nets effectively, as the local conditions can become very dynamic due to navigation-induced currents and wave action.

#### Trawl net

Similar to the strengths of larvae net technique, limited personnel are required for the operation of the trawl net. The net can be deployed while sailing without needing to be anchored, which is particularly advantageous in areas where anchoring is prohibited or impractical. Its adaptability extends to the possibility of monitoring plastic debris in different positions within the water column (surface, middle, bottom). One of the key strengths of this method is the high collection rate, enabling the retrieval of a significant number of samples per day. This, coupled with its ability to capture a wide variety of unique plastic categories, provides researchers with a comprehensive overview of plastic pollution in freshwater systems.

Despite their effectiveness in capturing plastic debris, trawl nets present several operational limitations. One common issue is the incidental collection of organic material as bycatch or entangled with the plastic items, making the plastic item identification process slower. Additionally, their utility may be limited by navigation constraints, as their large width during setup can impose spatial limitations on where they can be deployed.

The use of trawl nets presents several opportunities to enhance plastic pollution monitoring. With a small mesh size of 6 mm, these nets are capable of capturing small mesoplastics, thereby enhancing the effectiveness of the monitoring by capturing a wide range of plastic sizes. Moreover, their usage without anchoring in certain locations eliminates the need for permits in certain contexts, reducing bureaucratic obstacles. Additionally, trawl nets can serve as an indirect monitoring technique of fish populations, providing valuable insights into the species present in the ecosystem.

The implementation of trawl nets in environmental monitoring may be constrained by several threats, including practical and regulatory challenges. The use of trawl nets for environmental monitoring may be associated with high costs, including expenses related to vessel rental and personnel required for their operation. Additionally, regulatory constraints pose threats to the process, as obtaining permits for certain monitoring areas is often necessary.

#### Stow net

Likewise, limited personnel are required for the operation of the stow net. Stow nets offer a cost-effective solution, as they are relatively inexpensive compared to alternative methods, reducing overall project costs. Furthermore, these nets demonstrate efficiency in capturing larger pieces of plastic debris. Another notable strength is the low risk of clogging, ensuring continuous operation without frequent interruptions for maintenance.

Several weaknesses may affect their overall utility in riverine environments. For the deployment of this net, anchoring is required, which may not always be feasible or practical in certain rivers. Additionally, the large width of the setup can impose spatial limitations, constraining where the nets can be effectively deployed. Moreover, the depth of water also poses limitations, as stow nets may not be suitable for use in areas with shallow waters. Furthermore, stow nets cannot differentiate between depths in the water column, which can affect their ability to assess plastic pieces distributed across different layers. Concerning the efficiency, the used stow net could collect a low number of samples per day. Additionally, since part of the stow net had a mesh size larger than the target plastic size (e.g., > 5 mm), not all plastics that were present in the water column were collected, leading to lower catching efficiency in capturing smaller particles, such as meso- and microplastics.

Despite these constraints, the stow net method presents opportunities for interdisciplinary collaboration. The frequent bycatch of fish during deployments offers the potential to contribute to ecological monitoring initiatives, particularly when coordinated with fisheries research institutions. This dual-purpose application could enhance the value of fieldwork by supporting both plastic pollution assessment and biodiversity data collection.

Performing plastic monitoring in shipping lanes presents challenges due to the necessity of anchoring, which can be potentially dangerous in areas with high traffic density, posing safety risks to other ships. Furthermore, permits are required for using the stow net vessel. Regulatory constraints and requirements may pose threats regarding monitoring areas within the river.

### Applicability of sampling methods

The heatmap in Fig. 7 represents the level of applicability of each plastic sampling technique, offering a comparative visualization. In this context, a higher applicability score indicates that a method is more suitable, practical, and effective for application under the conditions of the present study, whereas a lower score suggests limited suitability. Among the methods, the trawl net showed the highest applicability score in the current study context. The adaptability factor, named here “Collection while sailing,” was a unique advantage of the trawl net. Conversely, the stow net method demonstrated the lowest applicability score.

## Discussion

Recent studies have described monitoring approaches and analytical procedures for sampling and assessing plastic pollution in river systems (Blettler et al., 2019; Owens & Kamil, 2020; He et al., 2024; Tasseron et al., 2024). Despite increasing research attention, only a few experimental studies have simultaneously compared different sampling techniques considering multiple factors (Obersteiner et al., 2025). To address this gap, our study examines the effect of monitoring techniques on the diversity, categorization, and absolute concentration of collected macro and mesoplastics.

The observed differences in the number of OSPAR categories related to collected macro- and mesoplastics pieces across different methods highlight variations in plastic diversity and categorization among methods. Notably, the trawl net demonstrated more unique categories compared to the larvae net for both macro- and mesoplastics. Similarly, when comparing the larvae net with the stow net, the latter exhibited a greater range of categories for both macro- and mesoplastics.

This low detectability of macroplastic categories is expected, since larvae nets (0.3 mm mesh) are typically used in studies targeting microplastics due to their finer mesh size (e.g., Di Mauro et al., 2017; Lahens et al., 2018; Yonkos et al., 2014), rather than larger macro- and mesoplastics debris. Nevertheless, despite the lower diversity of categories detected by the larvae net, certain categories were found in comparatively higher abundance using this method. This suggests that the stow net and trawl net may underestimate the presence of some (small) plastic types due to their larger mesh size.

A correlative study performed in the Elbe, Weser, and Ems Estuaries (southeastern North Sea) showed comparative results regarding the variety of categories found with the stow net method. In total, Schöneich-Argent et al. (2020) found 41 distinct categories, whereas Oswald et al. (2023) identified 51 different categories of macro- and mesoplastics classified according to the River-OSPAR classification system. The design of stow nets and net trawls typically covers a larger spatial area, increasing the likelihood of encountering a wider range of plastic debris, including rare or less abundant types. Therefore, to obtain a comprehensive understanding of the magnitude of plastic diversity in the riverine system, particularly regarding the impact of recent legislative measures on the presence of specific plastic categories, i.e., Directive (EU) 2019/904, it is imperative to use a monitoring technique capable of effectively collecting such data.

Despite the divergence in the number of OSPAR categories in relation to the collected macro- and mesoplastics pieces between different methods, there is a consistent pattern across the used methodologies. The most dominant category found during all monitoring campaigns was ‘Plastic film 2.5 −50 cm (soft)’ and ‘Plastic film 0—2.5 cm (soft)’ for macro- and mesoplastics, respectively. Similarly, earlier research in the Rhine found substantial proportions of unidentified fragments of plastic film and foils. The most dominant category, up to 83.5% of the total plastics, identified by de Vries (2023) was ‘plastic/polystyrene pieces of soft plastic’. Complementary, compositions of plastic pieces, in the water column, were also similar in a bend of the Nieuwe Maas River (distributary of the Rhine near the city of Rotterdam), investigated by Blondel & Buschman (2022) and in the Scheldt River, in Belgium investigated by Velimirovic et al. (2022), where the most identified plastics were unspecified fragments of soft plastic (foils), 91% and 88% of the amount of plastic debris, respectively.

Sampling methods also had a significant influence on the result with regard to the measured macro- and mesoplastics concentration. Sampling techniques showing high sensitivity, such as the larvae net, were better suited to quantify the concentration of smaller items, e.g., mesoplastics particles in the aquatic environment. Conversely, methods such as the stow and trawl nets are less sensitive to smaller debris but more effective in capturing a wider diversity of plastic categories and types. Overall, for macroplastic abundance, the larvae net yielded slightly lower, though not statistically different, concentrations compared to the stow net. These findings are consistent with previous work where different bioindicator species were selected based on their sensitivity and ability to reflect diverse plastic types in the environment. While the study from Valente et al. (2025) focused on fish species rather than sampling nets, a similar pattern has been observed, with highly sensitive indicators being more effective at detecting smaller or less abundant items, whereas polyspecific indicators capture a broader range of plastic types.

These variations emphasize the influence of the sampling method on the diversity of plastic types captured and underscore the efficiency and specificity of each method. For instance, critical aspects such as the selection of large mesh-size nets could affect the ability to catch smaller pieces of plastic, indicating a potential bias in the collected items (Oswald et al., 2023). Simultaneously, small mesh-size nets are effective for sampling microplastics, whereas due to their small frames, they may fail to sample larger macroplastics adequately, underestimating their abundance in the river (Blettler & Wantzen, 2019; Blettler et al., 2018; Lebreton et al., 2018; Weideman et al., 2020). For instance, a net with a 300 µm mesh size measuring 0.6 × 0.3 m, deployed at the water surface of the Orange-Vaal River System, South Africa, effectively collects plastics under flow velocities up to 3 m/s^1^. However, under lower flow conditions, turbulence around the net may cause some plastic items to bypass the net rather than being captured (Weideman et al., 2020).

When selecting the sampling method, it is essential to consider both the respective environmental conditions of the target area and the specific objectives of the study. Moreover, each of the methods presents distinct advantages and disadvantages that should be carefully weighed up in the context of the particular problem and boundary conditions. For instance, while net-based approaches may involve higher sampling and preparation costs, these can be offset by the benefit of collecting larger sample volumes and providing greater representativeness (Obersteiner et al., 2025). Therefore, carrying out a SWOT analysis, by identifying knowledge gaps (weaknesses), opportunities, strengths, and threats related to method development, highlighted the importance of considering mesh size, sampled volume, depth variation, and river morphology as an important step in method assessment. Further, factors related to logistics and resources available to ongoing policy processes and the scientific community are also pertinent (Montoto-Martínez et al., 2022). The larvae net showed advantages in terms of ease of deployment, depth differentiation, small width setup, and rapid sample processing. However, challenges related to anchoring, limited plastic category diversity, clogging risks, and sensitivity to flow conditions and navigation presence should be carefully considered when choosing this method for plastic monitoring in river environments. The small mesh size presents an opportunity for focused microplastic analysis, but the method's limitations should be weighed against its strengths and opportunities. The trawl net emerges as a versatile and powerful tool for plastic monitoring, offering advantages such as no anchoring requirements, adaptability to various water conditions, and the ability to provide detailed qualitative assessments. However, challenges related to the unintended capture of fish, limitations in highly navigated areas, high costs, and regulatory constraints should be carefully considered when opting for this technique. The stow net monitoring technique, while cost-effective and versatile, faces challenges related to anchoring, selective filtration limitations, and spatial limitations due to the large width of the setup. Opportunities lie in collaborative ventures with research institutions, while threats include regulatory constraints that may affect the technique's applicability in specific river environments. The balance between strengths, weaknesses, opportunities, and threats should be considered when determining the suitability of a method for plastic monitoring in rivers.

Although in this study it was possible to compare levels of plastic pollution obtained among different methods, a direct comparison between studies is challenging. Unfortunately, the lack of standardized methodology for sample collection and processing, along with divergent characteristics between rivers, could threaten the ability to accurately measure and compare data across different studies and regions (Lofty et al., 2023). Without uniform protocols, the true scale of plastic pollution remains unknown (Adomat & Grischek, 2021).

### Limitations

An in-depth analysis of the sampled volumes measured by the trawl net compared to the larvae net shows a 2.5 times higher discharge going through the trawl net when corrected for sampling surface area. This supports the hypothesis that the flow velocity meter used during the larvae net monitoring reports reduced volumes compared to ADCP measurements performed near the trawl net. When the concentration is corrected for the underreported sampling volume, the concentrations between the two methodologies become more consistent and remain within the same order of magnitude. Further limitations of this study involve operational constraints. Not all three nets can be deployed simultaneously from the same vessel due to space restrictions. In addition, vessel configurations required for operating the trawl and stow nets are not compatible. Consequently, direct comparisons among all three methods at the same location and time were not feasible.

## Conclusion

An effective monitoring strategy for plastic pollution is crucial for assessing its environmental impacts and guiding mitigation efforts. The results of this study revealed insights into the diversity, abundance, and unique characteristics of macro- and mesoplastics in riverine environments and indicated that recovery rates of macro- and mesoplastics vary significantly depending on the sampling technique. Among the evaluated approaches, the trawl net and stow net emerged as the most effective in collecting a diverse range of plastic categories, providing therefore a comprehensive characterization of the riverine plastic pollution, outperforming the larvae net. The trawl net, in particular, offered the most versatile and operationally robust option under the hydrodynamic conditions of the Rhine River. In contrast, the larvae net, although yielding higher concentrations of mesoplastics, systematically underrepresented larger debris categories. Similarly, the stow net’s inefficiency in capturing smaller particles points to potential underestimation of mesoplastics abundance. These differences highlight that each technique provides a partial perspective on the full plastic size spectrum.

A SWOT analysis is a useful tool to be considered when planning and budgeting for a net-based research initiative to ensure effective and compliant operations during environmental surveys and pollution monitoring efforts. Each sampling technique evaluated in this study presented distinct advantages and limitations regarding ease of use.

- Larvae nets offer ease of deployment, depth-resolved sampling, and strong sensitivity to small particles, but face challenges related to anchoring, clogging, navigational safety, and limited detection of larger debris.
- Trawl nets are versatile (i.e., able to collect samples while sailing) and efficient for both qualitative and quantitative assessments, capturing a broad diversity of OSPAR categories, yet they involve higher operational costs and regulatory constraints.
- Stow nets provide cost-effective long-term monitoring and broad category detection, but they have limited sensitivity to small particles and are spatially constrained by their fixed deployment (i.e., anchored vessels) and wide setup.

The observed variability among the techniques highlights the variability in performance and suitability among different sampling techniques and illustrates the dynamics of plastic pollution. Addressing these aspects requires careful consideration and an adaptive approach to ensure the effectiveness and applicability of the method. Additionally, it further emphasizes that the method selection must balance operational factors (e.g., mesh size, sampled volume, deployment feasibility), available resources, sampling efficiency, and the specific monitoring objectives and the environmental conditions of the target areas. Our results demonstrate that no single method is universally optimal for characterizing macro- and mesoplastics pollution. Instead, each technique operates within a clearly defined range of performance. Methods with high sensitivity do not necessarily capture the greatest richness of plastic categories, and vice versa. Furthermore, selecting the right combination of methods is essential to generate reliable, comparable data across studies and regions that can inform policymakers, lead to the development of effective mitigation strategies, and evaluate the progress under regulations, such as Directive (EU) 2019/904.

## Supplementary Information

Below is the link to the electronic supplementary material.

Supplementary file1 (DOCX 1255 KB)

## Data Availability

The datasets presented in this study were stored in the DANS EASY repository of the Radboud University, Nijmegen (10.17026/PT/AWUCI7).
