# Supplementary material for: Evaluation of riverine macro- and mesoplastic monitoring approaches
Source: Environ Monit Assess. 2026 Jan 16;198(2):134. doi: 10.1007/s10661-025-14889-4 (PMC12808205; doi:10.1007/s10661-025-14889-4)
Supplement: Supplementary file 1 — Supplementary file1 (DOCX 1255 KB) [file 10661_2025_14889_MOESM1_ESM.docx]

**Appendix A**

**
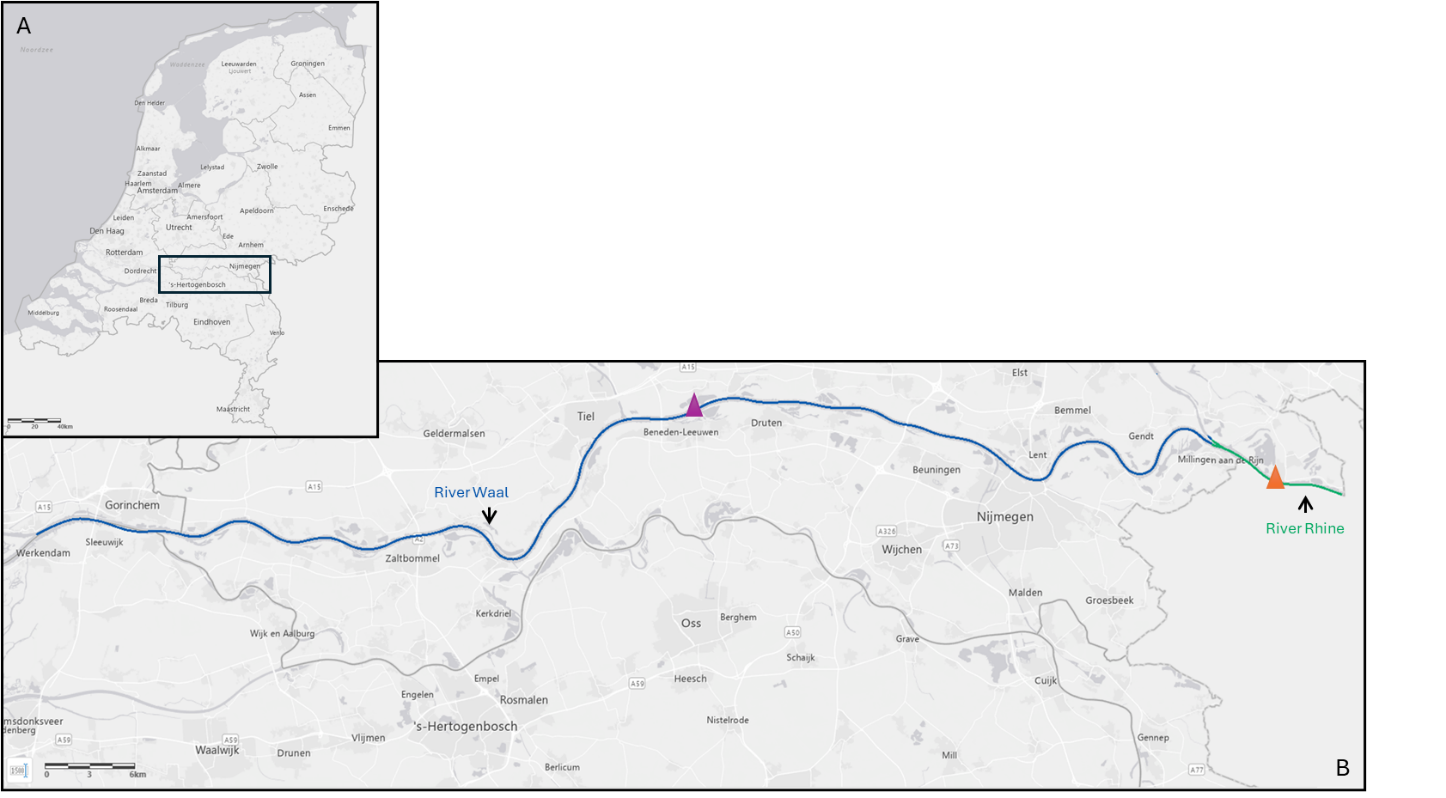
**

**Fig. A1.** (A) Map of the Netherlands including the study area and (B) sampling sites in the water column of the Waal River, with parallel plastic monitoring using larvae net and stow net (purple), and in the Rhine River with parallel plastic monitoring using larvae net and trawl net (orange).

**Appendix B**

**
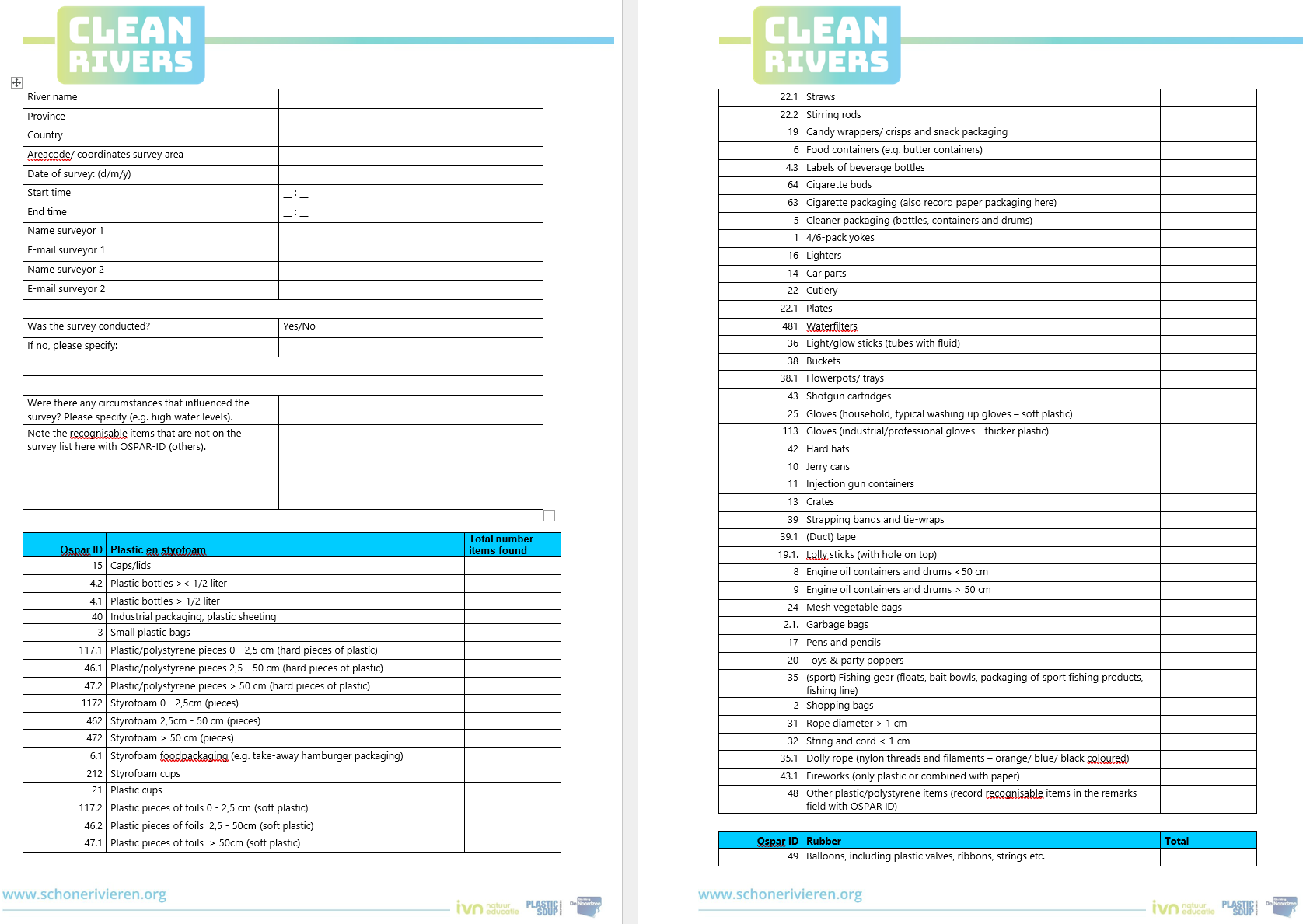
**

**
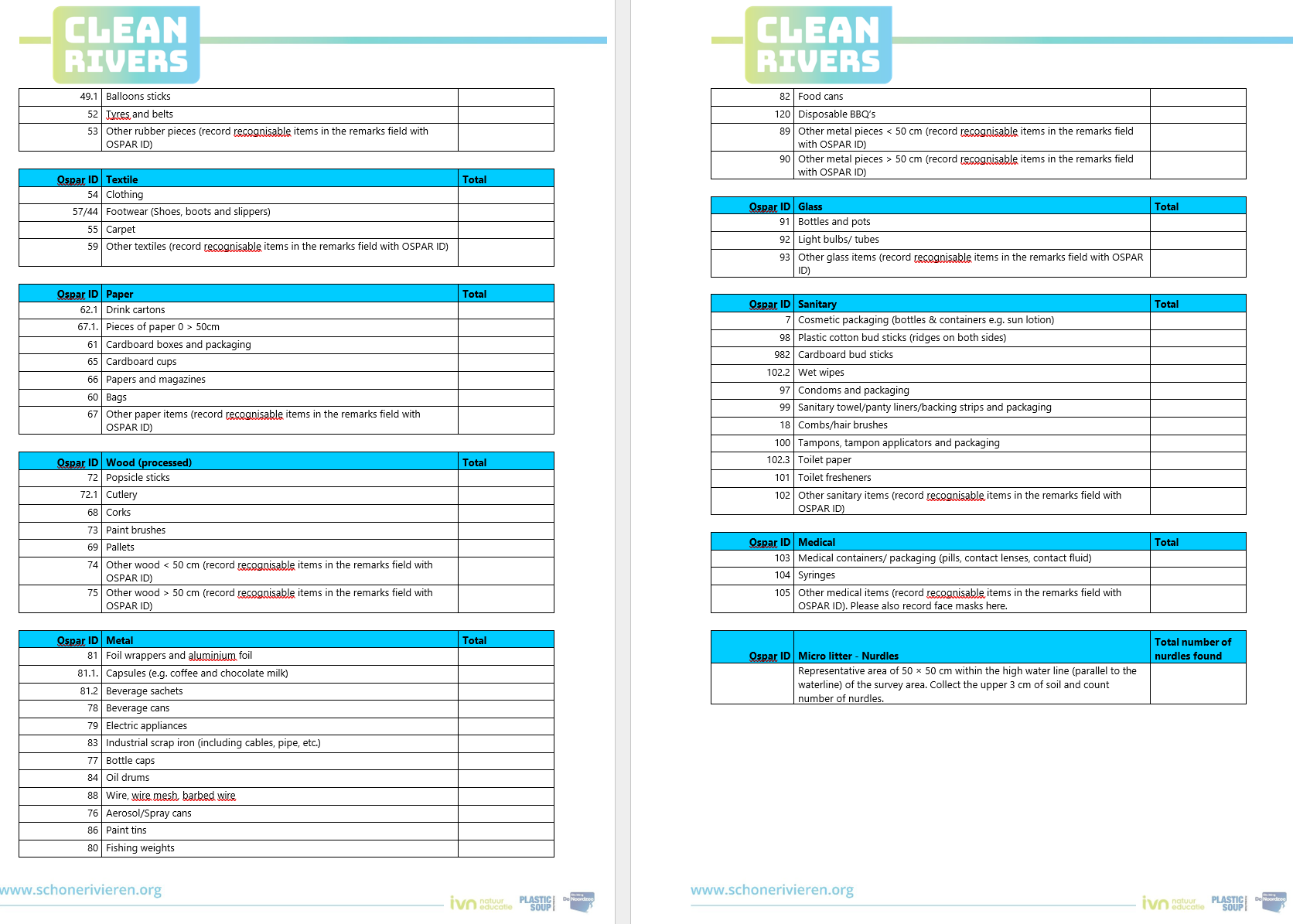
**

**Appendix C**

**Table C1.** Overview table of the factors considered to define the strengths, weakness, opportunities, and threats of each monitoring technique.

|  |  | **Methodology** | | |
| --- | --- | --- | --- | --- |
| **Parameter** | **Unit** | **Stow net** | **Larvae net** | **Trawl net** |
| Price per day | Monetary (euro's) | € 2,000 | € 2,900 | € 3,300 |
| Minimum № staff | Persons | 3 | 2 | 3 |
| Lab cost | - | Medium | High | Medium |
| Anchoring required | - | Yes | Yes | No |
| Collection while sailing | - | No | No | Yes |
| Width set-up (boat) | Meters | 6 | 1.5 | 5.5 |
| Width set-up (net) | Meters | 8 | 1 | 4 x 2 (left and right) |
| Spatial location | - | Yes^a^ | Yes^b^ | Yes^c^ |
| № of samples per day | Samples | 4 | 15 | 16 |
| Clogging risk | - | Low | High | Medium |
| Permits required | - | Yes | Yes^d^ | Yes^e^ |
| Depth differentiation | Meters | No | Yes | Yes |
| Fish bycatch | - | High | Low | Medium |
| Plant bycatch | - | Yes | Yes | Yes |
| Mesh-size | Millimeters | max 80 mm min 5mm | 0.3 mm | 6 mm |
| Selective filtration | - | Macro, meso | Micro, meso | Macro, meso, micro |
| ^a^ limited by depth and by navigation | |  |  |  |
| ^b^ limited by flow velocity and navigation | |  |  |  |
| ^c^ limited by navigation | |  |  |  |
| ^d^ for using shore structures | |  |  |  |
| ^e^ only when anchored |  |  |  |  |

**Appendix D**

**Fig. D1.** A) Relative abundance of macroplastic categories according to the River-OSPAR classification method during the larvae net and trawl net monitoring in the Rhine River in April 2022, and B) detailed observations of the differences in percentage between methods.


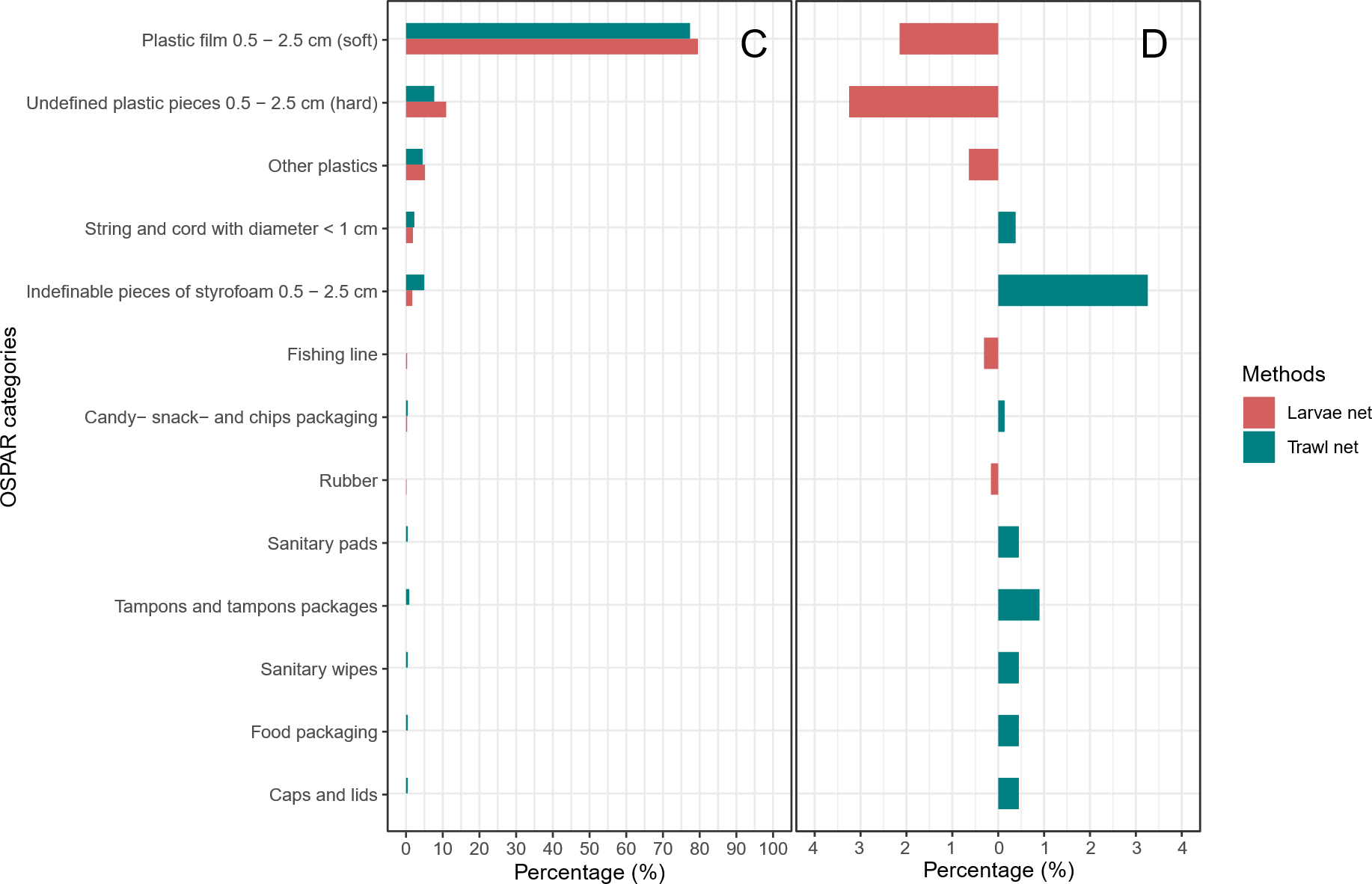


**Fig. D2**. C) Relative abundance of mesoplastic categories according to the River-OSPAR classification method during the larvae net and trawl net monitoring in the Rhine River in April 2022, and D) detailed observations of the differences in percentage between methods.


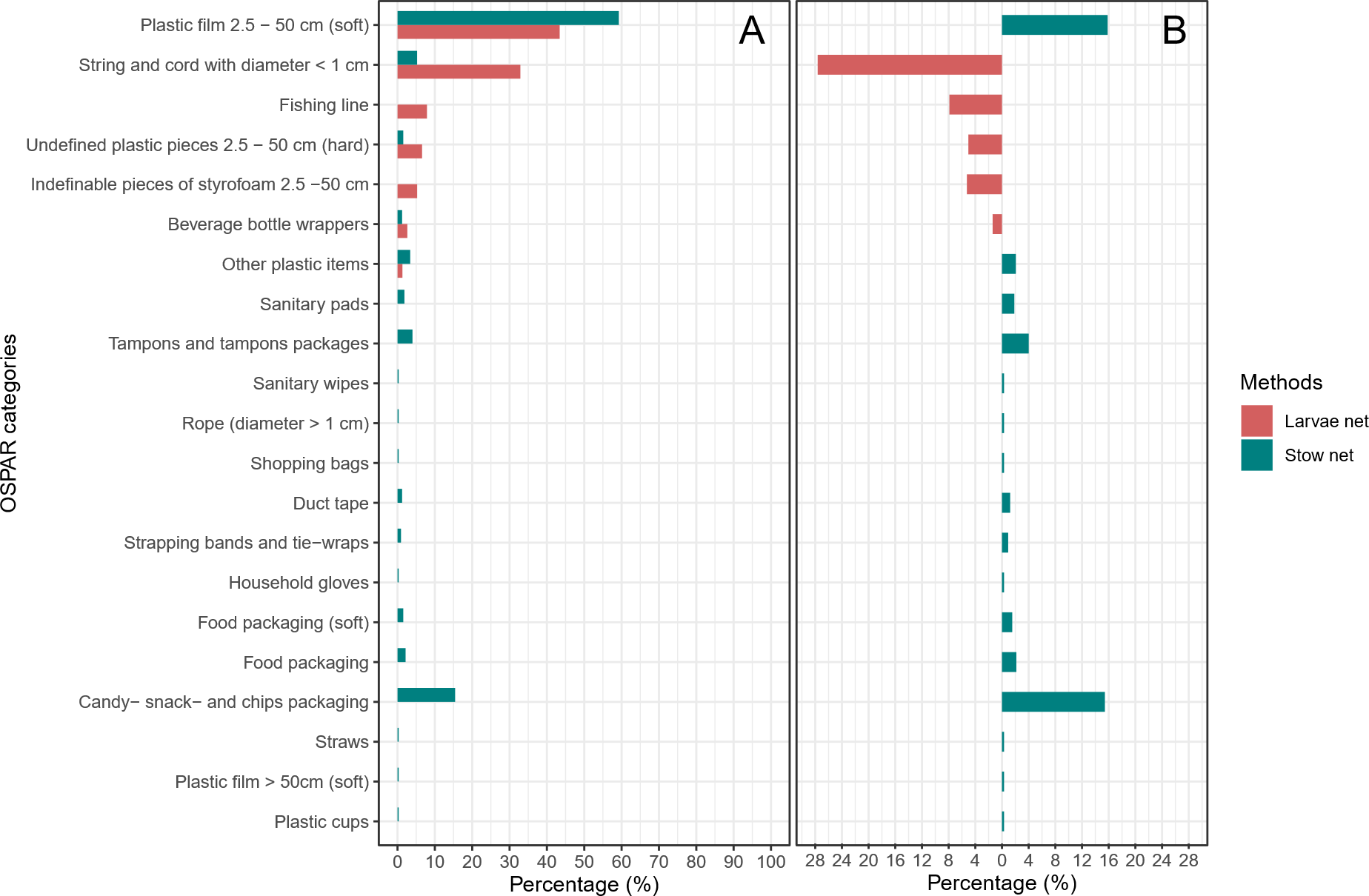


**Fig. D3.** A) Relative abundance of macroplastic categories according to the River-OSPAR classification method during the larvae net and stow net monitoring in the Waal River in October 2020, and B) Detailed observations of the differences in percentage between methods.

**Fig. D4.** C) Relative abundance of mesoplastic categories according to the River-OSPAR classification method during the larvae net and stow net monitoring in the Waal River in October 2020, and D) Detailed observations of the differences in percentage between methods.

**Appendix E**

**Table E1.** Macro- and mesoplastic concentrations (items/m³) during monitoring campaigns performed in the Rhine River using different methods, e.g., larvae net and trawl net

**Table E2.** Macro- and mesoplastic concentrations (items/m³) during monitoring campaigns performed in the Waal River using different methods, e.g., larvae net and stow net
